# Externalizing your eating disorder: a qualitative interview study

**DOI:** 10.1186/s40337-021-00486-6

**Published:** 2021-10-15

**Authors:** Marthe M. Voswinkel, Cleo Rijkers, Johannes J. M. van Delden, Annemarie A. van Elburg

**Affiliations:** 1 Altrecht Eating Disorders Rintveld, Wenshoek 4, 3705 WE Zeist, The Netherlands; 2 Eating Disorders Center, PsyQ Haaglanden, Lijnbaan 4, The Hague, The Netherlands; 3 Department of Medical Humanities, Julius Center for Health Sciences and Primary Care, University Medical Center Utrecht, Universiteitsweg 100, 3584 CG Utrecht, The Netherlands; 4 Department of Clinical Psychology, Utrecht University, Heidelberglaan 1, 3584 CS Utrecht, The Netherlands

**Keywords:** Anorexia nervosa, Externalization, Patient experience, Epistemic injustice

## Abstract

**Background:**

Anorexia nervosa (AN) is a psychiatric disorder with an ego-syntonic nature, causing many patients to perceive their AN as part of their personal identity. Therefore, an important part of treatment is the externalization of the eating disorder, in order to help patients to perceive AN as an external influence. Studies on patient experiences of externalization in treatment for AN are sadly missing. The aims of this study were to investigate, first, patients’ perspectives on the relation between identity and anorexia nervosa (AN) and second, their experiences of an externalizing approach during treatment.

**Method:**

A qualitative interview study was conducted including fourteen patients with AN in either Family Based Treatment, the Maudsley Model for Anorexia Nervosa Treatment for Adults, Specialist Supportive Clinical Management-Severe Enduring or Cognitive Behavioural Therapy-Enhanced.

**Results:**

There are important differences in participants’ perceptions on how AN is related to identity. AN was perceived as part of identity, as alien or as a different side of the self. Patients’ experiences towards an externalizing approach were ambivalent. Externalization was considered helpful, whilst also evoking a negative response. Participants reported feeling as if all their behaviour was referred to as part of AN, which elicited feelings of not being taken seriously or being wrongfully accused of being dishonest.

**Conclusions:**

First, there is considerable variation in the perceived relation between AN and identity. Second, an externalizing approach within treatment may lead to so called epistemic injustice. Awareness of these two facts is of importance for clinicians and the therapeutic relationship because that will help them to refrain from actions that can be perceived as epistemic injustice.

## Plain English summary

Many patients with anorexia nervosa (AN) have the experience that their eating disorder is part of who they are. Therefore, in the treatment of AN, healthcare professionals try to make clear to patients that there is a distinction between who they are and their AN. Through labelling certain thoughts and behaviour as stemming from AN, healthcare professionals guide patients in recognizing what is AN and what is not. This process is called externalization. In this study, fourteen patients with AN were interviewed on their thoughts about the relation between their identity and their eating disorder, and their experiences of externalization of AN within treatment. What becomes clear in these interviews is that patients’ perceptions on the relation between AN and their identity varies greatly, from experiencing AN as part of who they are to AN as something completely alien to them. Externalization is sometimes experienced as helpful, yet at the same time patients felt as if all behaviour was labelled as part of AN, which made them feel as if they were wrongly accused of being dishonest or not taken seriously. For healthcare professionals it is important to know that these side effects may arise when externalizing AN in treatment.

## Introduction

Anorexia nervosa (AN) is known for being a psychiatric disorder that is difficult to treat. Full recovery is reached in 46 percent of patients, about a third of patients show some improvement and in twenty percent of patients, AN becomes a chronic disorder [1]. Of all patients with AN, five percent dies from the disorder [2]. A possible reason for these low recovery rates is the fact that, often, AN has an ego-syntonic nature [3–5]. What characterizes this ego-syntonic nature is that patients will perceive AN as part of their true self, and not as an illness that requires treatment [6]. In contrast to most diseases or disorders, often, patients with AN do not experience an intrinsic wish to recover (mostly meaning: regain a healthy weight). Perceiving AN as part of the self could result in difficulties accepting treatment, sometimes actively resisting treatment, since treatment would entail dismissing part of the self [3]. The preoccupation with restrictive eating and weight loss, as well as the intense fear of eating and gaining weight, seems to leave little room for the former self. Therefore, the distinction between AN and the self can be unclear for patients [4, 7]. Within treatment, healthcare professionals (HCP’s) can guide patients in learning to recognize that AN is distinct from who they are. HCP’s attempt to clarify this distinction and one possible way of achieving this is by externalizing AN. The aim of externalizing a psychiatric disorder is to help patients to perceive the disorder as something separate, rather than as a part of the self [8]. Perceiving AN not as part of the self, but as an illness that has great negative influence on life and requires treatment, could help patients to accept treatment [9]. To externalize AN, HCP’s can use words to raise awareness in patients of the distinct nature of AN. For instance, HCP’s can ascribe certain behaviour to a healthy part of the patient, and ascribe behaviour that is evoked by AN to the ill part of the patient, or the illness. Another possibility to improve awareness of the difference between behaviour evoked by AN and healthy behaviour is helping patients to reflect on why they made certain decisions, and then choose which part of them, healthy or ill, was responsible for that decision.

From previous studies we know that patients suffering from AN have various ideas on the relationship between their identity and AN [4, 10]. Some patients experience AN as a part of who they are, whereas for others it is a distinct, external and ego-dystonic entity [4, 10]. Many patients experience a phenomenon described as the “anorexic voice”, a voice that is encountered as something internal, yet at the same time as something alien [11]. For those patients that experience difficulty to recognize a distinction between AN and identity, defining aspects of AN as disease or ill could feel problematic. Since these concepts are value-laden, describing something as a disease could be experienced as a negative judgement of the person. However, if a patient perceives AN as something external, by defining it as disease, this could give a patient the feeling of being supported in pursuing a shared goal: fighting AN. In Family Based Treatment (FBT) externalization is an important part of treatment which can help parents to be strict with AN and at the same time be a loving parent to their child [12]. Externalization in FBT may also help alleviate feelings of blame and guilt in parents [13]. The clinical impression however, is that a part of patients feel dismissed and patronised.

In treatment methods other than FBT, externalisation does not have the same integral role as it has within FBT. Therefore, within other treatment methods, whether externalization is used as an intervention, depends on whether the HCP decides to use it.

Previous studies into patient experiences with regards to the relation between AN and identity show great variation. On the subject how patients experience treatment for AN, many studies have been conducted and recently a qualitative metasynthesis has been published [14]. However, none of the studies has focussed specifically on the experience of an externalizing approach within treatment [5, 15–19]. One study on FBT discusses how parents experience externalization within treatment, but patients’ experiences were not investigated [13]. As externalization can play an important role within the treatment of AN, having insight in how patients experience this intervention is essential. The current study aims to elucidate these patient experiences.

Since how patients perceive the relation between AN and their personal identity could affect the experience of an externalizing approach, the aim of this study was twofold. The first objective was to investigate the variety of possible views regarding the relation between identity and AN. The second objective was to investigate patients’ experiences of this externalizing approach and the use of value-laden words in this approach.

## Methods

### Operationalization of identity

Within philosophy, psychology and neuroscience numerous theories exist on identity and the self [20]. Within the current study identity is operationalized as ‘narrative identity.’ In contrast to theories that describe the self solely as the centre of experience, the narrative self is a more substantial interpretation of the self [21, 22]. It is a result of the story we tell about who we are [22]. Through the story we tell ourselves, we try to make sense of our individual actions [23]. Multiple variations exist on the concept of the narrative self [22]. Overall, these variations can be distinguished based upon the function ascribed to the narrative and the elaborateness thereof. On the one hand, there are theories that suggest that the narrative is something that helps us understand ourselves and our actions. Other theories ascribe more importance to the narrative, by presenting it as something that actually shapes the self and identity [24, 25]. This last notion on the narrative self, known as ‘narrative identity’ is an elaborate story about who we are, what our values and goals in life are, and it is this narrative that constructs our identity [25]. In the current study, identity was operationalized as this elaborate notion on the narrative identity, in which the narrative constructs identity.

### Design

The objective of this study was to elucidate the experiences of patients suffering from AN with regards to the relation between AN and their identity, as well as their experiences of an externalizing approach within treatment. To this end an explorative qualitative interview study using inductive thematic analysis was conducted [26].

### Procedure

The study proposal was assessed by the Research Ethics Committee of the University Medical Center Utrecht, which judged that it was exempt from review under Dutch law. All participants were patients at Rintveld, Center for Eating Disorders at Altrecht. Informed consent was obtained from participants before the interview.

### Sample

All participants received treatment for AN at Rintveld, Center for Eating Disorders. To ensure that a wide range of experiences was captured, a maximum variation sampling method was used, with a focus on variation in treatment method, in- or outpatient treatment and duration of illness. Treatment methods included were Family Based Treatment (FBT) [12] often used for younger patients, the Maudsley Model for Anorexia Nervosa Treatment for Adults (MANTRA) [27] for older adolescents and adults, Specialist Supportive Clinical Management-Severe Enduring (SSCM-SE) [28] and CBT-E, Cognitive Behavioural Therapy-Enhanced [29]. FBT and MANTRA focus on recovery, whereas SSCM-SE focusses on quality of life. As stated in the introduction, externalization is an integral part of treatment within FBT. In MANTRA, SSCM-SE and CBT-E externalization is not a standard intervention. Participants were considered eligible if they were sixteen years or older, competent to consent, had been diagnosed with AN, as described in DSM-IV [30] or DSM-5 [31] and had been suffering from AN for at least one year. Since there often is a delay between start of symptoms and actual diagnosis, the criterium on illness duration was a subjective one. In patients who were diagnosed for less than a year, their subjective experience of the duration of AN symptoms before diagnosis was inquired. Patients with a subjective illness duration of one year or longer were considered eligible. BMI was not used as exclusion or inclusion criterium.

Participants were either recruited by the researcher (M.V.) in group therapy sessions, where an open invitation was extended after a short presentation on the study was given, or were informed by HCP’s engaged in their treatment. HCP’s at Rintveld, Center for Eating Disorders were informed about the study and the inclusion criteria. In the case of an eligible patient, they would inform them about the study. If patients were interested in participation, their name was given to the researcher, after which they were approached via e-mail to give additional information and to plan an appointment for giving informed consent and the interview.

### Data collection

In total, fourteen individual interviews were conducted by one researcher (M.V.). Interviews were either conducted on the premises of Rintveld or at participants’ homes. Interviews were held between May 2017 and January 2018 and lasted between 30 and 60 min. Topics in the interview focussed on the relationship between AN and what participants experienced as their identity, the role AN played in participants’ life stories and how AN influenced their lives and their choices. Participants were asked to describe how they viewed life with AN, their experiences of being treated for AN, whether they had ever come across externalizing language and how they experienced this approach. In "Appendix" an overview of the topics with examples of questions can be found.

The interviews were audio recorded after which the audiotapes were transcribed verbatim by one researcher (M.V.). To ensure anonymity, all participants were given a code in the audio tapes and in the transcripts. This code was only known to the researcher (M.V.).

### Data analysis

An inductive thematic analysis was conducted [32, 33]. Since the aim of the study was of an explorative nature, elucidating the variation in views and experiences of patients with AN, a thematic analysis was deemed the most appropriate type of analysis. During this study, the constant comparative method was used, meaning that, after the phase of the first four interviews, data analysis started whilst at the same time new interviews were conducted, allowing for the use of newly acquired data in the subsequent phase of the study [34].

Interviews continued until saturation was reached on the main themes of the study. Regarding the theme “AN in relation to identity”, saturation was defined as the point in data collection where the full spectrum, ranging from AN being identity or AN being inseparably intertwined with identity to AN being something alien to one’s own identity, was mentioned by the participants and new interviews did not add any new views on the relation between AN and identity. With regards to experiences of externalization, saturation was defined as that point in data collection where no new experiences, next to positive or negative experiences, ambivalence towards externalization, experiences related to being dismissed as a person, distrust or wrongful accusation, were reported by participants.

In the first phase of the analysis, transcripts of the first four interviews were thoroughly read and coded by M.V. and C.R., for which a bottom-up approach was used to construct the initial codes. Software program NVivo Pro 11 was used to support coding and data analysis. Where possible in-vivo coding was used, to reduce researcher interpretation and stay close to participants’ experiences. After coding the first four interviews, two researchers (M.V. & J.v.D.) revised the list of initial codes and constructed a coding tree, in which larger categories were identified. All interviews were thoroughly read and coded by two researchers (M.V. & C.R.) separately, after which the interviews and codes were reviewed by the same researchers. Differences in coding were discussed by the two researchers (M.V. & C.R.) until consensus on definitive codes was reached. When, during the process of coding the interviews, it was necessary to revise the coding tree or add new codes, the two researchers (M.V. & C.R.) discussed the necessary revisions and which codes to add. In order to obtain feedback on interpretations during data analysis, the data was discussed with two experts (professors medical ethics and eating disorders), after which central recurring themes were extracted from the data, defined and named. These themes were used in the descriptive model, as it is presented in the results section of this paper.

## Results

In total fourteen young women were interviewed. Characteristics of the study population are shown in Table 1. The main themes are described below and illustrative quotes are provided on these themes. Additional illustrative quotes can be found in Tables 2 and 3. Before turning to the results in more detail, one important general result of the study should be mentioned: all participants were remarkably capable of reflection on their diagnosis of AN and their experiences with regards to the relationship between AN and their identity.

**Table 1:** Characteristics of study population

Participant	Age	BMI	Symptom duration	Time since diagnosis	Treatment method	Inpatient/outpatient/day-treatment
A	22	13.7	12 years	11 years	MANTRA	Inpatient
B	18	19.3	2 years	1.5 year	FBT	Outpatient
C	22	18.5	7 years	2 years	SSCM-SE	Day-treatment
D	34	17.9	3 years	2 years	SSCM-SE	Day-treatment
E	20	16.8	6 years	5 years	MANTRA	Outpatient
F	25	17.9	10 years	10 years	SSCM-SE	Day-treatment
G	18	13.7	4 years	3 years	SSCM-SE	Day-treatment
H	17	18.3	2.5 years	2 years	FBT	Outpatient
I	19	16.0	2 years	1 year	MANTRA	Day-treatment
J	20	14.1	8 years	3 years	SSCM-SE/MANTRA	Day-treatment
K	19	14.7	4 years	8 months	MANTRA	Day-treatment
L	17	16.7	3 years	2 years	MANTRA	Inpatient
M	18	18.1	2.5 years	2 years	FBT	Outpatient
N	19	19.0	1.5 to 2 years	7 months	CBT-E	Outpatient

*BMI* Body Mass Index, *MANTRA* Maudsley Model of Anorexia Nervosa Treatment for Adults, *FBT* Family Based Therapy, *SSCM* Specialist Supportive Clinical Management, *CBT-E* Cognitive Behavioural Therapy-Enhanced

**Table 2:** Additional quotes on AN in relation to identity

AN is alien to identity
“It felt as something from the outside, something that imposed all kinds of rules on me.”—Participant F
“I would think “I couldn’t think clearly, so that could not have been me.” Or maybe it was, but then it was some kind of monster that had gotten into me.”—Participant M
AN has taken over identity
“The eating disorder has complete power over me, in such a way that my entire day is taken up by it.”—Participant D
*Participant F*: “It’s kind of my identity” *Interviewer*: In what way, your identity? *Participant F*: “Often, I do not know who I truly am, what I am good at, what I want in my life. And with my eating disorder, it’s a way of life, sort of.”
“At first, I was completely taken over by the eating disorder, because I was unaware of it.”—Participant K
“On the one hand I feel as if the eating disorder has taken over control, that it’s not up to me anymore. But on the other hand, my thoughts and thoughts belonging to the eating disorder are so severely entangled that I can no longer see it as two separate things.”—Participant L
“In the beginning I truly thought “I am my eating disorder,” and little by little, when I got better, it gradually became “I have an eating disorder.”—Participant M
AN is intertwined with and incorporated in narrative identity
“If I think of not having an eating disorder anymore, then I’m scared of feeling lonely, and that I would lose a great part of me. Because if feels like it’s something that I am, and something that belongs to me.”—Participant A
“For me it is something from the inside out … and it feels like it is a part of me. … And they say, ‘you have an eating disorder, it is not something that you are’, but I find it difficult, because it feels as if we have fused together”—Participant J
AN is an inauthentic side of identity
“It’s an inauthentic part.. because somewhere I know that it’s not me. … But over time it has gotten more and more closely connected to me. … It’s not a part of me … it’s just something that is clinging to me.”—Participant L
“I always thought that it was one entity, but I have come to realise that it’s actually two sides of me. … But there can only be one person in the end, so then it should be something inauthentic.—Participant E
AN is an authentic side of identity
“I think it’s two sides of me, two extremes”—Participant K
“It’s not something that I am, but it is something that belongs to me.”—Participant N

**Table 3:** Additional quotes on an externalizing approach

Positive experiences of an externalizing approach
*Interviewer:* “Did separating these two sides help you?” *Participant F:* “It feels okay to be able to say ‘Oh, that was my eating disorder.’ Or ‘that was truly the healthy side of me.’ I now know how to distinguish the two.”
“I really need other people to help me. They have to point it [the AN] out to me, because I have lost all track of my thoughts.”—Participant L
Ambivalence towards an externalizing approach
*Participant K:* Within treatment, they actually place the eating disorder in a chair, they really try to show that it’s something separate. And then they will point at the chair and say “that’s the one that is speaking now,” so they do really try to externalize it *Interviewer:* What’s that like for you? *Participant K:* In the short run, difficult. But if I’m motivated, then it’s fine, because then I know ‘it’s not me that’s wrong’ … They try to communicate it as clearly as possible, but sometimes I do still feel criticized as a person.”
Experiences of being dismissed as a person
“Some people say ‘I am not mad at you, but at your eating disorder, and I think that how you behave because of your eating disorder is unacceptable,’ whereas with other people I just have the feeling that they get angry with me … Then it feels as if they are attacking me as a person. Then I’ll think ‘Oh, they really do hate me’ “—Participant A
Experiences of distrust and wrongful accusation
“In all kinds of things he [participant’s father] saw something that originated from the eating disorder, even though that was not always the case. It felt as if I was wrongly accused of something.”—Participant J
“At a certain point it was the case that everything I said that wasn’t to their [participant’s parents] liking, was attributed to the eating disorder, while there were indeed things that I, myself, genuinely wanted or did not want. … I remember sometimes thinking ‘But this is just unfair’.”—Participant M

### AN in relation to identity

Participants’ stories on the relationship between AN and what they perceived as their identity varied widely. What becomes clear throughout the data is that different views on this relation can coexist and that how one views this relation is something that can change over the course of the disorder. This shift was mentioned by participants when asked to reflect on their view on the relation between AN and identity and whether this had evolved over the course of their illness. Analysis of the data in order to investigate whether the perception of the relation between AN and identity might be associated with patient characteristics, such as BMI, time since diagnosis or age of onset, shows no associations in this sample.

Participants’ views on the relation between AN and identity can be placed on a spectrum, ranging from AN being their identity, or being inseparably intertwined with identity as one end of the spectrum, to AN being something alien to one’s own identity at the other end of the spectrum.

“I have a strong sense that it is not who I am, that it just is something from the outside.”—*Participant I*

At the end of the spectrum where participants’ view AN as their identity, two different variants may be distinguished. Participants describe that at times when AN was most severe, often early in treatment or during relapses, they felt completely absorbed by AN and that it appeared to them as if they had become AN. In this variant it seems as if there is no room for a different narrative, other than the narrative of AN, which seems to have taken over the personal narrative, or has eliminated the need for a different narrative.

“It was so deep inside of me, it was so strong. At that point my body, and my mind as well, just didn’t feel like mine anymore.” —*Participant N*

Other participants describe AN as something that has gotten intertwined with, and incorporated in their identity. It seems that AN has gotten incorporated in the narratives, and is perceived as interwoven with identity.

“ ‘Who am I without an eating disorder?’ is not something I ask myself. I am with my eating disorder. Without it I don’t exist.” —*Participant C*

In the middle, between the two ends of the spectrum, perceiving AN as identity versus AN as something alien, a third alternative view on the relation between AN and identity can be recognized. This view encompasses the idea that AN is a different side of the person. Some participants describe AN as an authentic side of who they are, whereas others perceive it as an inauthentic side of themselves. This view of AN as another side of the person differs from the view held by participants that perceive AN as interwoven with their identity. The difference is in the degree of intertwinement described by participants. When AN was perceived as a different side of the person, it was described as something closely connected, yet at the same time as something separate from identity. Participants perceiving AN as their identity describe it as completely interwoven with identity. Among those participants who perceive AN as a different side of the person, some describe AN as being inauthentic to their true identity, as an entity that somehow had gotten attached to them, whereas other participants describe AN as an authentic, yet different side of their identity.

Upon looking at the proposed spectrum, one possible expectation could be that when participants experience AN as something alien to them, it would be experienced as an external entity. Another expectation could be that when a participant experiences it as something internal, it would consequently be experienced as a part of identity. Yet, this is not the case. What becomes apparent throughout the data is that participants that perceive AN as alien do not necessarily experience it as an external entity. The following quote by participant N illustrates this:

*“For me, AN truly is something that is on the inside.. But I wouldn’t say that it is a part of me. … In general, I don’t believe it is part of my identity, so in that way it does feel alien. ”*—*Participant N*

One explanation, given by participants, for why AN was perceived as something external but at the same time as something from the inside out, is the fact that AN affected their thoughts and their feelings. Participants perceived thoughts and feelings as part of what constitutes their mind, and since minds were experienced as something inside of them, AN was experienced as something from the inside out and not as an external entity.

### Experiences of externalization

All participants stated that they had come across language used to externalize AN, either by HCP’s within treatment or within the social sphere, by family-members or spouses. Questions such as “is this a choice out of personal taste or from your eating disorder?,” or statements such as “This is not the healthy you, this is your eating disorder,” were reported by many participants.

#### Ambivalence

The data on the use of language in order to help patients recognize AN as an illness and create a greater distance between person and AN indicate that participants hold ambivalent feelings towards this externalizing approach. A majority of participants described this approach, in helping them to view AN as something separate to them, as being helpful, yet difficult at the same time.

“Sometimes it [externalization] was all right, but sometimes it was quite annoying. It’s a good thing that they ask, but they ask it a lot. Not everything stems from your eating disorder. And especially on the inpatient ward, it varies between nurses, but some of them see everything, all your behaviour, as something from your eating disorder.”—*Participant J*

An important finding is that participants’ opinions on this approach were not static, but could differ per moment. Participants described it as being difficult in the short run, but they would also perceive it as necessary for recovery in the long run, since it could help them recognize which behaviour and which thoughts were evoked by AN. Some participants stated that it was helpful since they felt as if they needed someone else to clarify for them what belonged to AN and what not. Yet, at the same time, when it was pointed out that something participants thought, said or did was part of AN, it could be very confronting. When HCP’s referred to certain behaviour as diseased, this confronted participants with the fact that they were ill, which could cause irritation. Nonetheless, through referring to certain behaviour as part of a disorder, it was confirmed to participants that there was an actual illness, which could extenuate feelings of being personally responsible for AN.

#### Dismissed as a person

Almost half of participants in this sample reported that they perceived HCP’s attempts to separate AN from a healthy part of the person as a personal attack and therefore hurtful. It resulted in participants feeling as if they were being dismissed as an entire person, not solely AN.

“They [HCP’s] will say: ‘We’re not trying to destroy you, we’re trying to destroy your eating disorder,’ but it’s so close together, and it is in you so deep, that it does surely feel like they’re attacking you.”—*Participant N*

Participants reported difficulty in keeping in mind that it was AN that was being dismissed, rather than their ‘healthy’ selves. They explained this as an effect of the feeling that AN was so closely related to their identity, whereas others reported it having to do with the fact that they were still so absorbed by AN that they experienced difficulty in distinguishing AN from their selves.

#### Experiences of distrust and wrongful accusation

Another view towards an externalizing approach reported by about half of the participants was that they sometimes felt as if HCP’s, or family-members, referred to behaviour as part of AN when in their own opinion this was not the case; it could result in the idea that the person making the comment viewed everything the participant said as originating from AN. This was described as frustrating or sometimes even aggravating, and could result in a feeling of not being trusted or believed, or the feeling of being treated unfairly.

“I have gotten better at making these choices for myself, and when there is something that is truly me, then I get mad if people don’t believe me. Because I have thought about it deeply, and then they still say “But isn’t that your eating disorder?” Yeah, that’s obnoxious. … Then I tend to think: ‘Come on, believe me, it’s the truth.’ ”—*Participant E*

Some participants also mentioned that they felt as if they were not taken seriously, because of AN, which in turn could lead to frustration.

“My parents were taught at the clinic to distinguish the child from the eating disorder, and that not everything their child would say was her, that it could be eating disorder. So, quite often, if I did something stupid or behaved badly, my parents would say “Oh, just let her talk, it is the eating disorder speaking”. It would infuriate me, I’d think: ‘Do you really not take me serious anymore?’ “—*Participant M*

Participants reported that, initially, when someone referred to specific behaviour as part of AN, independent of whether this was a correct or incorrect observation, AN would take over control. They described feeling as if in that moment, they would become AN and mentioned that in that moment, it was extremely difficult to change the behaviour that led to the comment. Participant K said that, for her, this had to do with the fact that she felt as if she was already battling against AN, but it was still perceived as diseased behaviour by others. When she was confronted with this fact, she would turn towards AN, rather than change the behaviour at that time, since she felt like “it’s never good enough” (Participant K). Usually, it would be possible to reflect on that specific moment at a later time, and realise that it might indeed have originated from AN. This phenomenon was mentioned by many participants. The initial reaction of AN taking over control occurred independent of whether it was a HCP or a family-member making the comment. However, one participant did mention that for her, it was easier to accept that some behaviour was part of AN when it was a HCP that pointed this out to her, instead of a family-member.

## Discussion

The aim of this study was to explore how patients in different stages AN view the relation between AN and their identity, as well as investigate how patients experience externalization of AN within treatment.

The study shows that there are important differences in participants’ perceptions on how AN is related to their identity. They incorporate AN differently in their narrative and this can change over time, from being completely absorbed by, or intertwined with AN, to starting to view AN as something separate from their own identity. This shift was described by many participants during interviews, when asked whether they could remember having different views on the relation between AN and identity during the course of AN. As became clear in the results two subcategories can be distinguished within the group of participants who perceive AN as part of their identity. In the first subcategory, AN seems to have taken over the narrative identity, leaving no room for the old self. This occurs in patients who are in a severe stage of AN. Weight loss and malnutrition have psychological effects that reinforce further restricted eating and weight loss, leading to patients being drawn into a downward spiral: restricted eating has anxiolytic effects and decreases dysphoric mood, to some extent, whilst eating and weight gain heighten anxiety and dysphoric mood [35]. In order to suppress these negative feelings patients might restrict their intake again, resulting in weight loss and thus also a state of malnourishment. In turn this reinforces preoccupation with restrictive eating and weight loss, leaving no room for other thoughts than those caused by AN, which could result in a sense of AN taking over the narrative identity. This is a particularly difficult phase in recovery, since patients have to endure anxiety and distress to get to a point in their recovery process in which room for thoughts and (positive) feelings other than those related to AN will start to re-emerge, and with it parts of the person behind AN.

In the second subcategory within the group that perceives AN as a part of identity, AN has been completely incorporated in and entangled with the narrative identity. Reports from participants that state that they do not know who they are without AN, show us that AN could act as a substitute for an alternative narrative. Different views on the relation between AN and identity could be held by the same person at different times which shows that different views can coexist. Personal views on how AN related to identity could vary within the person, per moment and per situation. Possible relations between participants’ personal views regarding identity and patient characteristics were investigated, but not found in the sample of this study. Although patient perceptions on the relation between AN and identity were previously researched [4, 7, 10, 14, 15], this study adds to the existing literature as it operationalizes identity as narrative identity and places all varying experiences on a spectrum. What is consistent with previous research is the fact that views on the relation between AN and identity can change over time [4, 5]. With regards to the second objective of the study we can conclude that many participants have both positive and negative feelings about an externalizing approach in the treatment of AN. Participants experienced that, initially, AN takes over control in response to confrontation by HCP’s. Some participants expressed the sense of being dismissed as a person. This effect could be associated with the implicit value judgement words like illness, disease and disorder carry in them [36, 37]. In this sample, the participants that report having experienced externalization as a personal attack or feeling dismissed as a person, all reported being completely entangled with or absorbed by AN. However, this sample is very small to draw conclusions on whether patients’ view on the relation between AN and identity and the experience of externalization as an attack are associated. Important to report is the fact that even when participants had such negative experiences with externalization, they could still view it as an intervention that was necessary. The emerging themes in the data on an externalizing approach show that some participants have the feeling that all their behaviour is seen as part of AN, even when this is not necessarily the case. For some participants this could elicit frustration or aggression. These emotions are not necessarily negative. They could have a motivational effect and encourage a patient to change their behaviour. Yet, the experience of all behaviour persistently being ascribed to AN, without considering the patients’ own view on the explanation of their behaviour, and there thus being distrust because of AN, could be interpreted as a form of epistemic injustice.

Epistemic injustice is a type of injustice in which a person is harmed in her capacity as a knower, and is thus harmed in a very fundamental aspect of being human [38]. Epistemic injustice presents itself in different forms, but considering the results of this study, testimonial injustice is the most relevant form of epistemic injustice, since it is this form that could fit participants’ experiences. In many social encounters, a transaction of information occurs, usually through conversation between two persons, or in other words, testimony. The person listening has to judge whether the person that is speaking is trustworthy. These judgements are, however, subject to bias and affected by stereotypes and prejudice. If, because of prejudice, the person speaking is unfairly deemed untrustworthy, and is thus not believed, heard or taken seriously, this person is harmed in the capacity as a knower and testimonial injustice arises [38]. Patients in general, but psychiatric patients in particular, are more easily subject to this type of injustice than healthy people [39–42]. In the case of AN, the stereotypical image of a patient with AN is a patient who will take extensive measures to restrict intake or get extra exercise, these are core characteristics of the disorder [15, 43]. Yet, if patients with AN are automatically deemed untrustworthy due to the fact that the person listening is aware of their AN diagnosis, the judgement of whether one is trustworthy might be biased, due to this prejudice. If certain behaviour by a patient is referred to as part of AN by a HCP or a family member, and this happens automatically and without conscious deliberation, the judgement of whether the patient is trustworthy might be biased. The fact that some choice or behaviour might not be a direct consequence of AN could then be overlooked. Consequently, participants’ reports on the experience of being wrongly accused, not being believed or not being trusted could be interpreted as testimonial injustice. Therefore, when a patient with AN persistently encounters distrust or wrongful accusation, and this distrust is the result of an automatic response evoked by the awareness of the AN diagnosis and not the result of conscious deliberation, whilst also disregarding patients’ own views on the behaviour, this could be a case of epistemic injustice [38].

We acknowledge that it is impossible for HCP’s and family members to recognize correctly, in all instances, what is part of AN and what not. Therefore, completely preventing testimonial injustice from occurring may be impossible, and not referring to behaviour as AN and pointing this out to patients could have negative consequences on treatment outcome. Nonetheless, awareness of the possibility of bias in judgements of trustworthiness of patients with AN and reduce the subsequent automatic response through employing a more conscious and deliberate process before reaching a conclusion on whether a patient is trustworthy or not, could be a start in reducing unnecessary negative experiences of an externalizing approach.

Extensive literature on experiences with regard to an externalizing approach is missing, but previous research shows that patients with AN value a non-judgmental, respectful and supportive therapist [14, 44], as well as being seen as a whole person, rather than just a diagnosis [16, 19]. This study thus adds to the literature by elucidating patients’ experiences of an externalizing approach in the treatment of AN and from its results the tentative conclusion that such an approach may result in epistemic injustice, can be drawn. Our study had strengths and limitations. First, only female participants were included, since during recruitment no male participants were present in the attended therapy groups. Even though AN predominantly affects women, the lack of male participants is a limitation, as it is possible that male patients with AN could have different views and experiences regarding identity and externalization. In a follow-up study it would be interesting to include male patients and investigate whether their experiences differ from those of female patients. Also, looking back, in the analysis subtypes of anorexia, either restricting or purging, could have been taken into account, since that could result in different views on the relation between identity and AN. The heterogeneity of the sample could be seen as both a strength and a limitation. In a sample of 14 participants, heterogeneity is a limitation, since it complicates searching for possible links between participant characteristics and different views on the relation between AN and identity. However, by using purposive sampling and thus purposely generating a sample heterogeneous sample, the study managed to capture a wide variety of experiences, matching the objective of the study. Participants had varying durations of illness, age and treatment method, and were at different stages in their recovery process. Another strength was the fact that the primary researcher was not involved in treatment of the patients during the time of the study. This ensured an impartial investigation and gave participants a chance to speak freely of their experiences of treatment.

Further research is warranted to investigate whether patients’ views on the relation between identity and AN predict if they have a negative experience of externalization, as well as whether personal ideas about the relation between identity and AN affect treatment results in the long run. For an intervention as externalization, often used and powerful yet at the same time carrying a risk of causing negative side-effects, previous research is extremely limited. Therefore it is crucial to investigate its additional effects and side-effects on the therapeutic relationship and treatment outcome.

## Conclusions

This study’s results show that patients hold various views on the relation between AN and their identity and that these views can change over time. Awareness of this fact is of importance for HCP’s as it may help HCP’s realize that patients’ perspectives can evolve over time and thus during treatment. The way externalisation is used can be different in the early phases of treatment and in later stages; when an important focus is on exploring the person’s sense of self including the ownership of responsibility for managing one’s own feelings and vulnerabilities. Since a part of our patients feels dismissed and patronised, checking with patients what their perceptions are before launching in to externalising conversations and using language that is consistent with how they see and talk about things, could prevent those feelings. Within clinical practice this calls for an open and sensitive stance and flexibility in HCP’s, since different perspectives may require different approaches within treatment. With regard to an externalizing approach in the treatment of AN, this study’s results show that an externalizing approach has negative side-effects. First, an externalizing approach may result in an experience of negative value judgement in patients, and second, an externalizing approach can result in epistemic injustice. Within clinical practice awareness of these side-effects, and their possible causes, is of great importance for HCP’s since they could negatively affect the therapeutic relationship. An important implication for practice is that when using an externalizing approach, HCP’s should be aware of the existence of unconscious bias related to AN and the fact that this could cause epistemic injustice, since awareness of its existence reduces its negative effects, and could in turn reduce the prevalence of epistemic injustice as a result of an externalizing approach. Therefore, raising awareness of how an externalizing approach in the treatment of AN may cause epistemic injustice is a first step towards preventing it.

## Data Availability

The datasets generated and analyzed during the current study are available from the corresponding author on reasonable request.

## Appendix

**Table:**

Topic	Examples of questions
Experience of living with AN	*What would you tell someone without an eating disorder, if you would be asked to explain what it’s like for you to have anorexia nervosa?*
AN and its influence on ‘normal’ life	*What effect has anorexia on your life?* *How does anorexia influence your life?*
Meaning or function of AN	*Does anorexia nervosa have some kind of special meaning to you as a person?* *If so: can you tell me about this special meaning?*
Relation between anorexia nervosa and personal identity	*From previous research we know that people with AN can have different views on the relation between AN and their personal identity, from experiencing AN as their identity to viewing it as something completely alien, and there might be a lot of different views. Can you tell me something how you view the relation between AN and your identity?* *Do you feel like you have an eating disorder or if you are your eating disorder?*
Experiences of treatment in general	*What is treatment like for you?* *What is it like to be treated for anorexia?*
Externalization in treatment	*Have you ever come across people within treatment (e.g. healthcare professionals) that made a distinction between you and your eating disorder?* *If so:* *Can you give me an example?* *What was that like for you?* *Can you tell me something about what kind of effect this had on you?*
Externalization outside of treatment	*Have you ever come across people outside of treatment (e.g. parents, friends, family) that made a distinction between you and your eating disorder?* *If so:* *Can you give me an example?* *What was that like for you?* *Can you tell me something about what kind of effect this had on you?*

## Abbreviations

AN: Anorexia Nervosa
BMI: Body Mass Index
CBT-E: Cognitive behavioural therapy-enhanced
FBT: Family Based Treatment
HCP: Healthcare Professionals
MANTRA: Maudsley Model for Anorexia Nervosa Treatment for Adults
SSCM-SE: Specialist Supportive Clinical Management-Severe Enduring
